# Epidural Labor Analgesia Is Associated with a Decreased Risk of the Edinburgh Postnatal Depression Scale in Trial of Labor after Cesarean: A Multicenter, Prospective Cohort Study

**DOI:** 10.1155/2020/2408063

**Published:** 2020-01-16

**Authors:** Jing Sun, Yuci Xiao, Liwei Zou, Danyong Liu, Ting Huang, Zhao Zheng, Xuetao Yan, Aiwu Yuan, Yuantao Li, Xiaolei Huang

**Affiliations:** ^1^Department of Anesthesiology, Affiliated Shenzhen Maternity & Child Healthcare Hospital, Southern Medical University, Shenzhen 518028, China; ^2^Department of Obstetrics, Affiliated Shenzhen Maternity & Child Healthcare Hospital, Southern Medical University, Shenzhen 518028, China; ^3^Department of Anesthesiology, Bao'an Maternal and Child Health Hospital, Jinan University, Shenzhen 518106, China; ^4^Department of Anesthesiology, Longgang District Maternity & Child Healthcare Hospital of Shenzhen City, Shenzhen 518172, China

## Abstract

Postpartum depression is a disabling mental disorder commonly seen in parturients under trial of labor after cesarean, which causes serious harm to the parturients. The etiology is unclear. We hypothesized that epidural labor analgesia can reduce the incidence rate of postpartum depression. Enrolled multiparas were divided into the epidural labor analgesia group (*n* = 263) or nonanalgesia group (*n* = 160) according to their own request. Edinburgh Postnatal Depression Scale was used to assess their mental status at 48 hours and 42 days after delivery. Relative perinatal variables were collected and further analyzed using univariate analysis and multivariate logistic regression analysis to assess the relation of epidural analgesia with the occurrence of postpartum depression under trial of labor after cesarean. The Edinburgh Postnatal Depression Scale score 48 hours ≥ 10 in the no epidural analgesia group was 26.42% while the epidural analgesia group was 8.49% (OR, 0.209; 95% CI, 0.096–0.429; *P* < 0.001). The Edinburgh Postnatal Depression Scale score 42 day ≥ 10 in the no epidural analgesia group was 25.16% while the epidural analgesia group was 6.59% (OR, 0.235; 95% CI, 0.113–0.469; *P* < 0.001). The incidence of postpartum depression was significantly lower in the epidural labor analgesia group at 48 hours and 42 days. There was also a significant relation between the Edinburgh Postnatal Depression Scale scores at 48 hours and 42 days after delivery. Epidural analgesia, discomfort within 42 days, and self-rating anxiety scale are independent predictors of postpartum depression for trial of labor after cesarean in 42 days. Epidural labor analgesia is associated with a decreased risk of postpartum depression. Further study with a large sample size and more centers is needed to evaluate the impact of epidural analgesia on the occurrence of postpartum depression. Chinese Clinical Trial Register, ChiCTR-ONC-17010654.

## 1. Introduction

Postpartum depression (PPD) is a disabling mental disorder usually occurring within 12 months after maternal production, which causes serious harm to both the mothers and families [1]. PPD may cause not only depression, insomnia or lethargy, significant weight loss or increase in weight, psychomotor disturbance or developmental delay, feeling of no value and excessive guilt, decreased self-esteem, and difficulty concentrating but also in extreme cases, self-harm, suicide, or infanticide [2–4]. In addition, it may lead to some childhood and adolescent developmental and behavioral problems to the children [5, 6]. Studies have shown that the incidence of PPD among parturients is about 15% to 30% in China, and it receives increasing attention with the implementation of universal two child policy in China [7]. It is reported that many women of childbearing age opt to have a second or third child, 32.7–50% of which underwent cesarean sections when giving birth the first child [8–11]. Following a cesarean section, the alternative modes for subsequent labor include repeated cesarean section (RCS) and the trial of labor after cesarean (TOLAC). Recent data show that TOLAC is the more effective delivery method because it costs considerably less expense than RCS while also receives better results in reducing the risk of postpartum hemorrhage (PPH) and pelvic adhesions [12]. However, although parturients get better treatment insurance with TOLAC and have experience of giving birth, they are equally prone to anxiety, fear, and tension, even more serious than the first time which is related to their large expectation of the sex and health of the fetus because of the unique culture of preference of boys to girls in China [13]. These factors are prone to PPD and cause harm to parturients. But, the etiology of PPD is complex of which the main risk factors include a history of psychiatric illnesses, mood instability during pregnancy, marital disharmony, poor social support, and stressful life events. Specifically to parturients, the intensity of labor pain was also a factor related to mood disorder in the early postpartum period [14]. The study of Eisenach et al. also confirmed that the severity of acute postpartum pain predicted the occurrence of PPD [15]. Ding Ting et al. reported 4 months after delivery, there was a lower incidence rate of PPD in parturients who received epidural and paracervical blockade during vaginal delivery [16]. In contrast, another study examining PPD rates among 1326 women did not demonstrate a difference between women with intrapartum epidural and those without [17]. Besides the conflicting results in the literature, the subjects of previous studies on PPD are almost aiming at nulliparous women rather than pluripara; therefore, further study is warranted.

We speculated that epidural labor analgesia could decrease the incidence of PPD in TOLAC, especially the vaginal birth after cesarean (VBAC). This study was to investigate correlation of epidural labor analgesia in TOLAC with a decreased risk of PPD.

## 2. Materials and Methods

### 2.1. Ethics and Informed Consent

The study, which complied with the Helsinki Declaration and relevant Chinese clinical trial research regulations, was approved by the Ethics Committee of Affiliated Shenzhen Maternity & Child Healthcare Hospital, Southern Medical University, Bao'an Maternal and Child Health Hospital, Jinan University, and Longgang District Maternity & Child Healthcare Hospital of Shenzhen city. Written informed consent was obtained from all participants. This study was registered in the Chinese Clinical Trial Register (registration number: ChiCTR-ONC-17010654), which participated in the World Health Organization International Clinical Trials Registry Platform.

### 2.2. Study Design and Patient Recruitment

This multicenter prospective, observational cohort design was conducted in three tertiary hospitals in Shenzhen, China, including Affiliated Shenzhen Maternity & Child Healthcare Hospital, Southern Medical University, Bao'an Maternal and Child Health Hospital, Jinan University, and Longgang District Maternity & Child Healthcare Hospital of Shenzhen city between February 2017 and February 2018. Inclusion criteria were as follows: multiparas with singleton fetus in the vertex presentation at ≥35 weeks, no systemic analgesics were used currently, and eligible for TOLAC after being assessed by an obstetrician. Parturients with the presence of epidural labor analgesia contraindications were excluded from the study. On admission to Labor Analgesia Consultation Clinic during 34-35 weeks of prenatal, all eligible parturients were informed about the study and signed the consent. According to ACOG Practice Bulletin, [18] labor analgesia could be performed as long as regular uterine contractions began and the parturient required analgesia, so in this study, each parturient made a decision by herself to have epidural labor analgesia or no pain relief at all and then were divided into two groups accordingly: epidural analgesia group, i.e., experiment group (*n* = 263); no epidural analgesia group, i.e., control group (*n* = 160). Other forms of analgesia were not available during labor.

### 2.3. General Data Collection

*Baseline Data*. Specific information was collected (Table 1). The mental status of parturients was assessed using the Zung Self-Rating Anxiety Scale (SAS) and Social Support Rating Scale (SSRS). SAS was used to evaluate the anxiety and SSRS was used to assess maternal social support, and final scores were calculated and are presented in Table 1. Perinatal data of parturients were recorded (Table 2). Neonatal information including gender, birth weight, and Apgar score at 1 and 5 minutes after delivery was recorded. Apgar score is a standard assessment method for checking the physical condition of a child immediately after birth. A score of 10, 7 or less, and below 4, respectively, indicate normal newborns, mild asphyxia, and severe asphyxia [19].

### 2.4. Epidural Labor Analgesia

All parturients undergoing TOLAC were observed in the delivery room and underwent continuous electronic fetal monitoring. Vital signs including blood type, electrocardiogram (EEG), hemoglobin (HGB), completing count, and coagulation function were monitored and further assessed whether the parturients were suitable for TOLAC which together with mode of delivery induction of labor or oxytocin and mode of delivery were decided by board certified obstetricians.

For the epidural analgesia group, epidural labor analgesia was administered entering the labor process in the delivery room after cervix examination and contraindications exclusion. Parturients received a standard protocol for labor epidural analgesia. After entering the delivery room, the peripheral venous access of parturients who were experiment group was opened, the lateral recumbent position was taken, and epidural puncture (AS-E epidural catheterization kits) was performed through L2-3 or L3-4 intervals, and the epidural catheter was inserted at a depth of 3–4 cm. First, the test dose of 3 ml of 1 : 200,000 epinephrine + 1.5% lidocaine was injected and observed for 3–5 min to exclude the catheter into a body vessel or subarachnoid space, and then the drug with 10 ml of 0.1% ropivacaine mixed solution was administered by the epidural catheter once. After observing for 30 minutes, if there were no obvious adverse reactions such as hypotension, nausea, and vomiting, an analgesic pump could be connected. The analgesic pump capacity was 130 ml, and the drug was 0.08% ropivacaine + 0.4 ug/ml sufentanil. The ZZB-I pulse type analgesic pump was used, and the parameter setting was as follows: the pulse frequency was one pulse per hour, the dose was 10 ml, the injection rate was 400 ml/h, the PCA (patient-controlled analgesia) dose was 8 ml, and the locking time was 30 min. Epidural labor analgesia was used until the baby was delivered. It was performed by a certificated anesthesiologist, and anesthetic volume was adjusted until the visual analog scale (VAS) pain score was under 4. If the parturient could not tolerate the pain or the VAS pain score was higher than 5 during labor, she could require rescue analgesia therapy. Rescue analgesia was with 8 ml of 0.67% lidocaine. Blood pressure is measured every 5 min during the first 20 min and hourly during the continuous patient-controlled analgesia usage. VAS pain scores were recorded 30 minutes after the epidural loading dose. The specific approach of VAS was drawing a 10 cm horizontal line on the paper. One end of the horizontal line was “no discomfort” in Chinese, the other end was “worst discomfort imaginable” in Chinese, and the middle part indicated different degrees of pain. We let the patient draw a mark on the horizontal line according to the feeling of self, indicating the degree of pain [20]. Anesthesiologists and anesthesia nurses of the three hospitals, who participated in this study, were staying in the delivery room 24 hours a day and trying their best to provide a “full” and “complete” truly painless delivery to every woman who received labor analgesia. For the control group, no analgesics were administered and VAS pain scores were recorded during the delivery.

### 2.5. Postpartum Assessment

The level of depression was assessed at 48 hours and 42 days after delivery using the Edinburgh Postnatal Depression Scale (EPDS) [21, 22]. In this study, PPD was defined as an EPDS score of 10 or higher at 42 days after delivery. Primary outcome was EPDS at 48 hours and 42 days after delivery. The parturients were asked to complete the EPDS 48 hours after delivery and received a call and family visit in 42 days later as a part of a larger follow-up study. All the above questionnaires were completed by parturients themselves, without discussing answers with their families. In the meanwhile, VAS was recorded as an indicator of the level of discomfort within 42 days after delivery. The VAS pain score measurement during labor, the postpartum assessments, and other data collection were performed by five unified training investigators who were not blinded to the type of analgesia but did not participate in the patient care.

### 2.6. Sample Size Calculation and Statistical Analysis

In previous years, the three hospitals had a labor analgesia rate of about 50 percent; we assumed that the number of patients in each group was equal. PPD was treated as a binary outcome. According to the published literature, we assumed that the incidence of PPD would be 25% in the nonmedicated parturients and 10% in the parturients who received analgesia [23–25]. The calculated sample size that would provide 80% power to see this difference based on a 2-tailed significance level of 0.05 is about 200 patients. Considering the rate of loss of follow-up, the sample was amplified by 10%, and the final minimal sample size was 287 patients. The sample size calculation was performed by using 2 independent proportions' power analysis on PASS 2008 (Kaysville, UT).

Continuous variables are presented as mean ± standard deviation (SD) or median (interquartile range). Data were compared with the use of *t*-test or Wilcoxon rank sum test. Categorical variables are presented as number of patients (percentage). Data were analyzed with the use of *χ*2 test or Fisher's exact test. The association between the use of epidural labor analgesia and the occurrence of PPD was assessed with univariate logistic regression and multivariate logistic regression analysis. Propensity score matching (PSM) was used in this study to validate the results of logistic regression that reduce the potential selection bias. Two-sided *P* < 0.05 was regarded as significant. All data were entered and analyzed in R (version 3.5.0).

## 3. Results

### 3.1. Baseline Maternal Demographic

**Figure 1:**
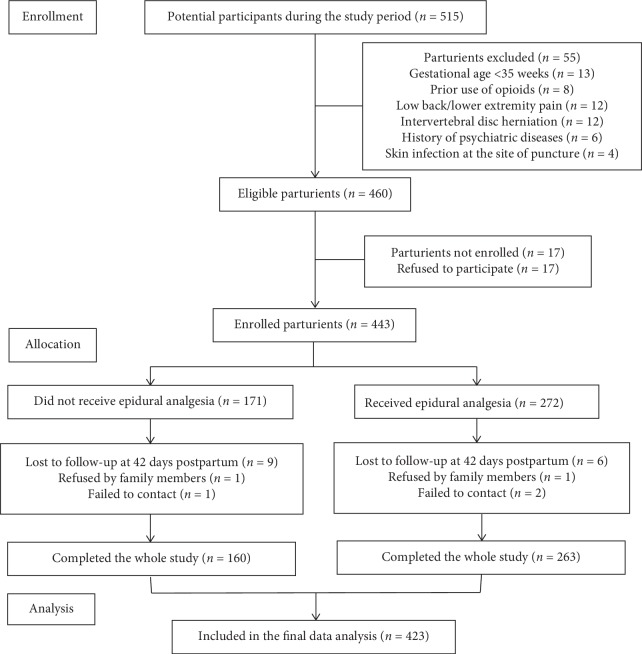


A total of 515 pregnant women who opted TOLAC were screened for eligibility, and 55 women were excluded based on criteria (Figure 1). 17 of enrolled parturients did not complete the whole study. In the end, 423 in total were included in the analysis, among which 263 were given epidural analgesia while 160 were not. 225 cases in the epidural analgesia group underwent VBAC and 38 cases underwent RCS. The characteristics of the study participants are shown in Table 1.

### 3.2. Primary outcome: Occurrence of Postpartum Depression

The incidence of EPDS ≥ 10 at 48 hours after delivery in control and experiment group, respectively, was 42 of 159 (26.42%) vs 22 of 259 (8.49%) under TOLAC. It was significantly lower in parturients who received epidural labor analgesia than those who did not receive epidural labor analgesia (*P* < 0.001).

The incidence of EPDS ≥ 10 at 42 days after delivery in control and experiment group, respectively, was 40 of 159 (25.16%) vs 17 of 258 (6.59%) under TOLAC. Obviously, the parturients who received epidural labor analgesia had a lower incidence of EPDS ≥ 10 at 42 days after delivery (*P* < 0.001).

In addition, there was a significant correlation between the EPDS scores at 48 hours and 42 days after delivery (Pearson correlation coefficient = 0.657, *P* < 0.001).

### 3.3. Effects of Epidural Labor Analgesia

36 (1.6%) had VAS pain score above 5 at 10 minutes (after the first loading dose). No parturient had a VAS pain score above 5 at 30 minutes (after the supplemental dose). The VAS pain scores at 6 cm and 10 cm cervical dilation were significantly lower in those who received epidural analgesia than in those who did not. The effects of labor analgesia evaluated by parturients themselves were good in 243 cases (89.67%), fair in 23 cases (8.49%), and poor in 5 cases (1.84%). The results of other perinatal and neonatal variables are described in Tables 2 and 3.

### 3.4. Factors Associated with the Occurrence of Postpartum Depression

When PPD at 48 hours was used as a dependent variable, univariate logistic regression analyses revealed that 7 of all the recorded parturient and neonatal variables were significant (*P* < 0.05) (Table 4) for TOLAC. They were epidural analgesia, hospital name, family income, SAS, SSRS, platelets (PLT) (125–350), and perineum/cervical laceration. Multivariate logistic regression analysis identified 5 independent predictors; they were epidural analgesia, hospital name, SAS, SSRS, and PLT (125–350). Epidural analgesia during labor was significantly associated with a decreased risk of depression at 48 hours after delivery for TOLAC (OR, 0.209; 95% CI, 0.096–0.429; *P* < 0.001) (Table 4).

Furthermore, propensity score matching was used to reduce the potential selection bias and validate the results again. Whether or not “Epidural analgesia” was used as the dependent variable and other factors were used as the independent variables. We used 1 : 1 nearest neighbor matching selects for 107 individuals in the control group and 107 individuals in the analgesia group (66 unmatched). After matching, the logistic regression was performed. Univariate analysis was performed using the outcome of PPD at 48 hours (depression or no depression) as the dependent variable, and whether or not “Epidural analgesia” and “other confounding factors” as the independent variables. Factors with *P* < 0.05 were included in the multivariate model, and stepwise regression was performed according to the AIC (Akaike information criterion) minimum criteria. 4 independent risk factors were obtained, and they were epidural analgesia, SAS, SSRS, and PLT (125–350); (Table 5).

**Figure 2:**
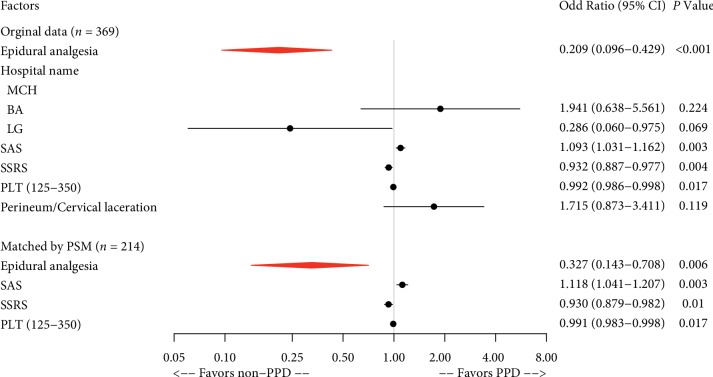


Therefore, the common independent influencing factors of PPD at 48 hours in the TOLAC population before and after propensity matching were epidural analgesia, SAS, SSRS, and PLT (125–350) (Figure 2).

When PPD at 42 days was used as a dependent variable, univariate analyses revealed that 9 of all the recorded parturient and neonatal variables were significant (*P* < 0.05) (Table 6) for TOLAC. They were epidural analgesia, anxiety and depression during pregnancy, unplanned pregnancy, changes in marital relationship during pregnancy, SAS, SSRS, HGB (g/L: 115–150), perineum/cervical laceration, and discomfort within 42 days. Multivariate logistic regression analysis identified 3 independent predictors, and they were epidural analgesia, SSRS, and discomfort within 42 days. Epidural analgesia during labor was significantly associated with a decreased risk of depression at 42 days after delivery for TOLAC (OR, 0.235; 95% CI, 0.113–0.469; *P* < 0.001) (Table 6).

Furthermore, propensity score matching was used to reduce the potential selection bias and validate the results again. Whether or not “Epidural analgesia” was used as the dependent variable and other factors were used as the independent variables. We used 1 : 1 nearest neighbor matching selects for 107 individuals in the control group and 107 individuals in the analgesia group (66 unmatched). After matching, the logistic regression was performed. Univariate analysis was performed using the outcome of PPD at 48 days (depression or no depression) as the dependent variable, and whether or not “Epidural analgesia” and “other confounding factors” as the independent variables. Factors with *P* < 0.05 were included in the multivariate model, and stepwise regression was performed according to the AIC minimum criteria. 4 independent risk factors were obtained, and they were epidural analgesia, SAS, SSRS, and discomfort within 42 days (Table 7).

**Figure 3:**
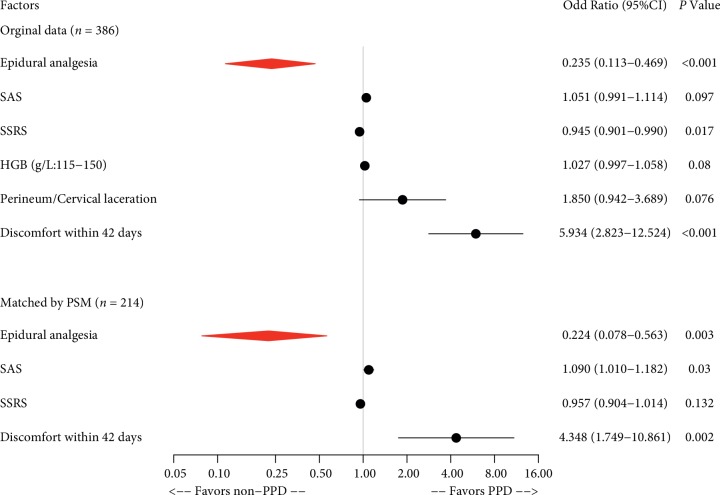


Therefore, the common independent influencing factors of PPD at 42 days in the TOLAC population before and after propensity matching were epidural analgesia and discomfort within 42 days (Figure 3).

### 3.5. Effect of Prediction of PPD for TOLAC in 48 Hours

**Figure 4:**
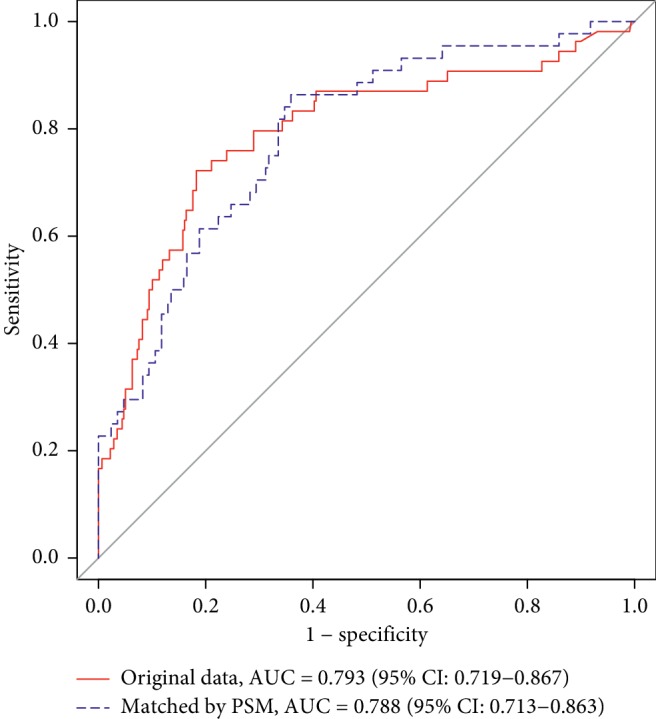


We use the two multivariate logistic regressions to predict PPD in TOLAC. The model without PSM showed that AUC (area under the curve) = 0.793, 95% CI: 0.719–0.867, sensitivity = 0.722, specificity = 0.818, positive predictive value = 0.402, and negative predictive value = 0.945. The model with PSM showed that AUC (area under the curve) = 0.788, 95% CI: 0.714–0.863, sensitivity = 0.864, specificity = 0.641, positive predictive value = 0.384, and negative predictive value = 0.948 (Figure 4).

### 3.6. Effect of Prediction of PPD for TOLAC in 42 Days

**Figure 5:**
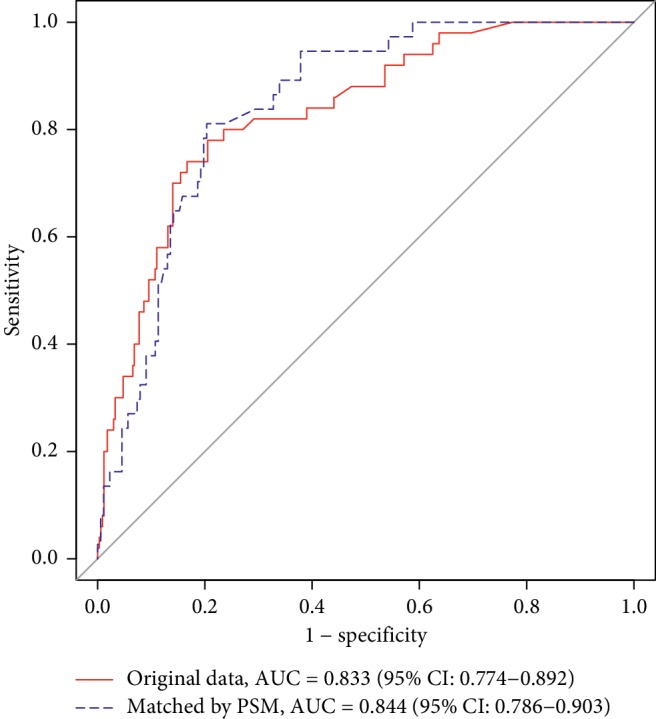


We use the two multivariate logistic regressions to predict PPD in TOLAC. The model without PSM showed that AUC (area under the curve) = 0.833, 95% CI: 0.774–0.892, sensitivity = 0.780, specificity = 0.795, positive predictive value = 0.361, and negative predictive value = 0.960. The model with PSM showed that AUC (area under the curve) = 0.844, 95% CI: 0.786–0.865, sensitivity = 0.811, specificity = 0.797, positive predictive value = 0.455, and negative predictive value = 0.953 (Figure 5).

## 4. Discussion

In the study, the incidence of PPD was reported from 20.3% to 29.5% aiming at nulliparous [23, 25], whereas the incidence rate of pluripara in this study was 13.5% in TOLAC which is much lower than that of the nulliparous, and reasons are not clear.

This study drew the conclusion that epidural labor analgesia is correlative with a decreased risk of EPDS in TOLAC, whereas the discomfort within 42 days, SAS score, and a high EPDS score early after delivery were associated with increased risks of EPDS. In the experimental group, more parturients received VBAC than RCS, which may cause the low incidence of high EPDS scores. The cause of PPD is often complex and has many influencing factors [26, 27]. For most women, labor is inevitably accompanied by intense pain and stress [28]. There were some studies which reported a correlation of the level of labor pain and the risk of PPD [14, 15]. Moreover, the labor pain evaluated in the early postpartum period (from 36 hours to 3 days postpartum) was found to be associated with PPD [14, 15]. In our study, the labor pain was evaluated during labor at 5 stages and the pain scores were significantly lower in parturients who received epidural analgesia than those who did not (Table 2), and corresponding incidence rate of PPD is relatively lower. In this study, although remifentanil could rapidly cross the placenta into the fetal circulation, we did not use remifentanil for the method of application of remifentanil may be associated with the impairment of neonatal outcome [29]; the effects of labor analgesia was evaluated by parturients themselves, and 243 cases (89.67%) felt good, 23 cases (8.49%) felt fair, and 5 cases (1.84%) felt poor. Labor analgesia can minimize pain of pregnant women, provide humanized medical services to pregnant women, help parturients establish confidence in natural labor, improve the rate of natural childbirth, reduce the adverse effects of pain on mother and baby, increase the rate of eutocia, increase blood flow to the placenta, improve fetal oxygen supply, relieve adverse reaction of labor pain, reduce or eliminate the parturients' childbirth pain, reduce maternal unnecessary physical consumption, maintain the dignity of parturients' childbirth, and let parturients enjoy the joy of child labor [30–32]. The overall painless labor analgesia rate of this study was 62.17%. And Affiliated Shenzhen Maternity and Child Healthcare Hospital had a painless labor analgesia rate of 57.74%, Bao'an Maternal and Child Health Hospital had a painless labor analgesia rate of 98%, and Longgang District Maternity and Child Healthcare Hospital of Shenzhen City had a painless labor analgesia rate of 47.17%. The painless delivery rate of Bao'an Maternal and Child Health Hospital was so high, mainly because the hospital fully implemented the “painless hospital” construction and responded to the call of the World Health Organization to “improve the rate of painless delivery,” as long as there was no contraindication to labor analgesia in the spinal canal after entering the delivery room. All women underwent painless delivery. If the mother refused to give birth painlessly, she could only go to other hospitals to give birth.

Besides the pain variables, the independent predictors of PPD were anxiety and depression during pregnancy [33], perceived stress, number of past stressful life events, lack of social support, history of depression, childhood maltreatment, and maternal nativity status. There is ample evidence that shows PPD has adverse effects on mothers, infants, and their families [34–37]. Mothers with depression are slow and insensitive in their interactions with children and show a higher incidence of negative behaviors such as smoking and not using car seat belt [38, 39]. Mothers not only care about physiological needs of infants but also significantly influence the cognitive and social development. Therefore, it is not surprising that maternal depression is related with low cognitive and increased behavioral problems in infants and children. Emotions are often felt in the body (anxiety as stomach “butterflies,” grief as “heartache”) and associated with specific topographical body sites of sensations. Therefore, in this study, we used the discomfort within 42 days to describe the pluripara state and interestingly found that there was a close association between EPDS score and discomfort. Many parturients experienced varying degrees of discomfort located in the perineum, vagina, and breasts or severe headaches after giving birth. Many discomforts were not serious, but had an adverse effect on maternal mood. This suggests that the discomfort cannot be ignored. Physical discomfort can cause mental changes, such as PPD. Women with PPD are exquisitely sensitive to the massive gonadal steroid withdrawal that occurs at birth. This biological vulnerability interacts with predelivery risk factors for major depressive disorder (MDD) [40]. In a model including nearly 2000 female twins and analyzing occurrence of MDD lasting over a 1 year, 52% of the variance of MDD was explained by 3 factors: [41] internalizing (early life onset of anxiety disorders), externalizing (conduct problems and substance abuse), and adversity (childhood maltreatment and interpersonal problems). SAS was used to evaluate the anxiety within 2 weeks before delivery which reflected internalizing factors [42]. SSRS assessed the social support situation of women [43]. In the current study, SAS score was a risk factor while SSRS score was a protective factor, which was consistent with existing literature.

The postpartum hemorrhage rate (defined as < 500 mL) was reported as 2%. This is extremely low. It may be because the Grade Three Class-A hospitals perform strict evaluation and monitoring of TOLAC maternity and carry out 1‐on‐1 midwifery. The exact reason why platelets become meaningful variables is unknown and can be verified by further clinical studies or animal experiments. In the epidural labor analgesia group, initial time of lactation was shorter, which was completely the benefit of labor analgesia for parturients, and labor analgesia reduced labor pain and reduced the release of stress hormone catecholamines so that the prolactin level was increased [44, 45]. About the influencing factor of episiotomy, the incident rate of it in the experiment group was higher than that of control group obviously, and the difference was statistically significant; it may be related to the extension of the first stage and the second stage. In this study, the time of the first stage and second stage in the experiment group was indeed longer than the control group, but the time was within the normal range, which is shown in Table 2. For the second stage of women with epidural labor analgesia, as long as the mother and infant were safe, the time of labor could be up to 3 hours [46]. Professor Shapiro, an obstetrician at Harvard Medical School, once said that as long as the mother and infant are safe, waiting is a virtue for the second stage. In this study, the time of labor was longer, but vaginal delivery was successful, which was exciting. At the same time, in the analysis of logistic regression of labor time in PPD, there was no statistical significance, which indicated that the labor time and episiotomy did not increase the incidence of PPD. The influential factor of perineum/cervical laceration was still statistically significant at 48 hours and 42 days after delivery in single factor analysis and multivariate regression analysis, which could be seen in Figures 2 and 3. Interestingly, after performing propensity matching and control of confounding factors, it was not statistically significant. This could be explained that this variable was a covariate and did not act as an independent variable for PPD but acted as a risk factor with other variables.

In univariate analysis, participation in maternity classes was a meaningful variable, *P* < 0.05. Although some hospitals started maternity schools early, the participation rate of pregnant women has not been high. In recent years, with the population of “eugenics policy” and the promotion of the benefits of painless delivery, the participation rate of maternity schools has increased significantly. This study focused on women who had given birth to a second child after a cesarean section; the second production method after cesarean section, breastfeeding (China used to mislead mothers that milk was more nutritious), postpartum care, psychology consultation, painless delivery, and so on are hot topics of concern for multiparous. So in this survey, the participation rate in maternity schools was higher than that in western countries. The rates of attendance at childbirth classes during pregnancy were 33.5% and 46.4% in the two groups, and there were statistically significant differences.

At present, there is no uniform screening time point for PPD, and the two commonly used screening time-points are within 4 weeks after delivery (Diagnostic and Statistical Manual of Mental Disorders‐IV) and within 6 weeks after delivery (International Statistical Classification of Diseases and Related Health Problems‐10) [47]. In addition, it has been suggested that PPD should not be screened in the first few days after delivery because symptoms are not fully developed [48]. However, high scores of antepartum or peripartum depression are strong predictors of PPD. In our study, EPDS was assessed at 48 hours and 42 days after delivery and the 42-day EPDS score was used for diagnosing initial screening of PPD. This study also found that a high early EPDS score was an independent predictor of PPD. It should be noted that most multiparous women delivering vaginally would have gone home by 48 hours in the UK which is different in China. The mother delivering vaginally can only be discharged from hospital 48–72 hours after delivery. During this period, the maternal blood routine, bleeding, urination, and lochia discharge were mainly observed. More importantly, in China, newborns can only be injected with Bacillus Calmette–Guerin after 48 hours, and the plantar blood can be collected after 72 hours. Therefore, the time of discharge from the hospital in China is also different from that in Western countries.

There were several limitations of this study. First, the parturients were grouped according to their decision. Second, analyzed laboring women were of the same ethnicity. The effect of region, culture, or ethnicity could not be excluded. Third, the diagnosis of PPD was not performed by psychiatrists. Although EPDS could be used to examine PPD, the effectiveness of Chinese parturients has been well confirmed [49–51]. Fourth, an observational study could not determine whether there was a causal relationship between epidural labor analgesia and the decreased risk of PPD. Last, the follow-up time was not long enough. New episodes of depression occur in 14.5% of women in the first 3 months after birth, and the 1-year period prevalence was a striking 21.9% (95% CI, 15.1%–30.0%) [52]. If the follow-up time was prolonged to one year, the occurrence of the PPD might be higher. It could be of help to know the deeper effect on PPD.

## 5. Conclusion

It was found that epidural analgesia during labor may be associated with a decreased risk of PPD. Further study with a large sample size and a longer follow-up time is clearly needed to evaluate the impact of epidural analgesia on the occurrence of PPD.

## Figures and Tables

**Table 1:** Baseline maternal demographic and obstetric characteristics of parturients who completed the study.

Characteristic	TOLAC (CTRL)		TOLAC (EXP)		Statistical value	*P* value
	*N* (all)	*n* (%) or mean ± SD	*N* (all)	*n* (%) or mean ± SD		
Mode of delivery	160		263			<0.001
Normal delivery		111 (69.38%)		225 (85.55%)		
Cesarean		49 (30.63%)		38 (14.45%)		

Hospital name	160		263		41.021	<0.001
MCH		131 (81.88%)		179 (68.06%)		
BA		1 (0.62%)		59 (22.43%)		
LG		28 (17.50%)		25 (9.51%)		

General information						
Age (year)	160	32 ± 4	262	32 ± 4	21481.5	0.667
Gestational age (day)	158	272 ± 14	260	274 ± 10	18846.5	0.157
BMI (kg/m^2^)	158	26.45 ± 2.93	259	26.59 ± 3.08	19040.5	0.234
Maternal education >12 y	116	116 (72.96%)	193	193 (74.23%)		0.819
Husband education >12 y	118	118 (74.21%)	207	207 (79.62%)		0.228
Housewives	159	13 (8.18%)	259	21 (8.11%)		1
Family income (¥/mo)^*∗*^	146		249		0.696	0.706
≤10,000		21 (14.38%)		29 (11.65%)		
10,001–20,000		53 (36.30%)		90 (36.14%)		
>20,000		72 (49.32%)		130 (52.21%)		

History of pregnancy and childbirth						
Gravidity					2.264	0.322^$^
2	70	70 (44.59%)	98	98 (37.69%)		
3	62	62 (39.49%)	121	121 (46.54%)		
>3	25	25 (15.92%)	41	41 (15.77%)		

History of depression and trauma	158	0 (0.00%)	262	4 (1.53%)		0.302

Impact of childbirth on work or re-employment	158	10 (6.33%)	260	14 (5.38%)		0.672

Maternity leave time	157		258			0.031
No		1 (0.64%)		6 (2.33%)		
Legal time		93 (59.24%)		178 (68.99%)		
Full-time		63 (40.13%)		74 (28.68%)		

Anxiety and depression during pregnancy	158	66 (41.77%)	262	87 (33.21%)		0.094

Cigarettes, alcohol, long-term medication	158	2 (1.27%)	261	1 (0.38%)		0.56

Whether the husband is satisfied with the baby's sex	158	144 (91.14%)	260	236 (90.77%)		1

Caring health knowledge during pregnancy						
① Routine obstetric examination	158	151 (95.57%)	261	251 (96.17%)		0.801
② Attendance at childbirth classes during pregnancy	158	53 (33.54%)	261	121 (46.36%)		0.011
③ Learn about parenting through cell phones or books	158	132 (83.54%)	261	224 (85.82%)		0.573

Unplanned pregnancy	158	62 (39.24%)	262	90 (34.35%)		0.346

Whether the maternal is the only child in one's family	158	17 (10.76%)	260	36 (13.85%)		0.449

Changes in marital relationship during pregnancy	157	2 (1.27%)	260	1 (0.38%)		0.559

SAS	156	32.88 ± 6.20	261	31.14 ± 5.89	23757	0.004

SSRS	156	45.74 ± 7.27	261	46.27 ± 5.82	19937.5	0.723

MMSE	156	28.15 ± 1.13	261	28.10 ± 1.06	21158.5	0.483

Prepartum Laboratory test						
HGB (g/L:115–150)	159	119.28 ± 15.05	259	116.68 ± 11.17	22643	0.087
PLT (125–350)	159	209.47 ± 55.51	260	210.03 ± 53.70	20399.5	0.822

BMI: body mass index; SAS: Self-Rating Anxiety Scale; SSRS: Social Support Rating Scale; MMSE: mini-mental state examination; HGB: hemoglobin; PLT: platelet; ¥: Chinese Yuan; SDP: standard deviation. ^$^Pearson's chi-squared test; ^*∗*^total income of husband and wife. Comparisons were made using Student's *t*-test or Wilcoxon rank sum test for nonnormally distributed variables. Comparisons were made using Pearson's chi-squared test and Fisher's exact test for proportions.

**Table 2:** Perinatal variables of parturients who completed the study for TOLAC.

Variable	TOLAC (CTRL)		TOLAC (EXP)		Statistical value	*P* value
	*N* (all)	*N* (%) or mean ± SD	*N* (all)	*N* (%) or mean ± SD		
Mode of delivery	160		263			<0.001
VBAC		111 (69.38%)		225 (85.55%)		
Cesarean		49 (30.63%)		38 (14.45%)		

Instrumental delivery in VBAC	27		43			1
Vacuum extraction		15 (13.51%)		23 (10.22%)		
Forceps		12 (10.81%)		20 (8.89%)		

Episiotomy	149	42 (28.19%)	256	111 (43.36%)		0.003

Perineum/Cervical laceration	149	70 (46.98%)	255	106 (41.57%)		0.3

Uterine rupture	160	0 (0%)	263	0 (0%)		1

Estimated blood loss after delivery (mL)	154		260			1
≤500		151 (98.05%)		255 (98.08%)		
>500		3 (1.95%)		5 (1.92%)		

Labor duration in VBAC						
The first labor duration (min)	108	334.14 ± 225.94	221	526.93 ± 266.85	6050.5	<0.001
The second labor duration (min)	102	28.09 ± 31.62	217	46.14 ± 32.64	5836	<0.001
The third labor duration (min)	108	8.62 ± 3.87	222	9.43 ± 5.03	11211.5	0.294
Initiating lactation period (hours)	157	11 ± 13	262	7 ± 12	24276.5	0.002

VAS pain at epidural (cm)						
T0: time 0	99	6.34 ± 2.16	219	8.20 ± 1.19	5191.5	<0.001
T1: PCEA after 30 min	100	7.00 ± 2.16	217	0.94 ± 1.61	21103	<0.001
T2: cervical:6 cm	100	7.67 ± 2.33	211	1.09 ± 1.71	20404.5	<0.001
T3: cervical:10 cm	99	8.64 ± 2.13	210	1.78 ± 2.00	20213	<0.001
T4: immediate delivery of the fetus	97	1.65 ± 2.44	209	1.88 ± 1.60	8062	0.002

Feeding patterns within 48 hours	158		233			0.361
Breast		152 (96.20%)		218 (93.56%)		
Milk		6 (3.80%)		15 (6.44%)		

Feeding amounts within 48 hours	147		230			0.491
Normal		137 (93.20%)		219 (95.22%)		
Discomfort within 42 days	159	33 (20.75%)	256	28 (10.94%)		0.007

EPDS score 48 hours						<0.001
<10	117	117 (73.58%)	237	237 (91.51%)		
≥10	42	42 (26.42%)	22	22 (8.49%)		

EPDS score 42 days						<0.001
<10	119	119 (74.84%)	241	241 (93.41%)		
≥10	40	40 (25.16%)	17	17 (6.59%)		

Comparisons were made using Student's *t*-test or Wilcoxon rank sum test for nonnormally distributed variables. Comparisons were made using Pearson's chi-squared test and Fisher's exact test for proportions.

**Table 3:** Neonatal variables of parturients who completed the study for TOLAC.

Neonatal outcomes	TOLAC (CTRL)		TOLAC (EXP)		Statistical value	*P* value
	*N* (All)	Mean ± SD or *n* (%)	*N* (All)	Mean ± SD or *n* (%)		
Weight	148		250			0.903
<3500		114 (77.03%)		190 (76.00%)		
≥3500		34 (22.97%)		60 (24.00%)		
Admission to neonatal ward after birth	153	12 (7.84%)	256	20 (7.81%)		1
1 min Apgar	159	0 (IRQ)	261	0 (IRQ)	12485.5	0.535
5 min Apgar	159	0 (IRQ)	261	0 (IRQ)	12542	0.292

Data are mean ± SD, or *n* (%). Comparisons were made using Student's *t*-test or Wilcoxon rank sum test for nonnormally distributed variables. Comparisons were made using Pearson's chi-squared test and Fisher's exact test for proportions.

**Table 4:** Univariate and multivariate analysis of PPD in TOLAC 48 hours after delivery.

Variable	Univariate		Multivariate (*n* = 369)	
Independent	*P* value	OR (95% CI)	*P* value	OR (95% CI)

Epidural analgesia	<0.001	0.259 (0.145–0.449)	<0.001	0.209 (0.096–0.429)

Hospital name				
MCH		1 (reference)		
BA	0.705	0.862 (0.377–1.785)	0.224	1.941 (0.638–5.561)
LG	0.049	0.299 (0.071–0.856)	0.069	0.286 (0.060–0.975)

General information				
Age (year)	0.816	0.992 (0.923–1.066)		
Gestational age (day)	0.253	1.017 (0.991–1.050)		
BMI (kg/m^2^)	0.38	0.958 (0.869–1.049)		
Maternal education >12 y	0.301	0.736 (0.416–1.338)		
Husband education >12 y	0.235	0.694 (0.386–1.293)		
Housewives	0.713	1.190 (0.430–2.821)		
Family income (¥/mo)^*∗*^				
≤10,000		1 (reference)		
10,001–20,000	0.057	0.475 (0.222–1.039)		
>20,000	0.011	0.382 (0.183–0.817)		

History of depression and trauma	0.086	5.645 (0.667–47.762)		

Impact of childbirth on work or re-employment	0.456	1.476 (0.475–3.838)		

Maternity leave time				
No		1 (reference)		
Legal time	0.987	2517827.629 (0.000–NA)		
Full-time	0.987	3841697.685 (0.000–NA)		

Anxiety and depression during pregnancy	0.115	1.543 (0.896–2.642)		

Cigarettes, alcohol, long-term medication	0.408	2.770 (0.128–29.334)		

Whether the husband is satisfied with the baby's sex	0.187	2.259 (0.781–9.581)		

Caring health knowledge during pregnancy				
① Routine obstetric examination	0.287	0.531 (0.178–1.948)		
② Attend maternity classes	0.062	0.581 (0.323–1.015)		
③ Learn about parenting through cell phones or books	0.354	0.720 (0.369–1.498)		

Unplanned pregnancy	0.052	1.706 (0.992–2.923)		

Whether the maternal is the only child in one's family	0.743	1.138 (0.497–2.367)		

Changes in marital relationship during pregnancy	0.05	11.226 (1.060–243.732)		

SAS	<0.001	1.092 (1.045–1.142)	0.003	1.093 (1.031–1.162)

SSRS	<0.001	0.895 (0.858–0.932)	0.004	0.932 (0.887–0.977)

MMSE	0.377	1.118 (0.872–1.431)		

Prepartum Laboratory test				
HGB (g/L: 115–150)	0.085	1.017 (0.998–1.037)		
PLT (125–350)	0.002	0.992 (0.987–0.997)	0.017	0.992 (0.986–0.998)

Initiating lactation period (hours)	0.273	1.011 (0.990–1.030)		

Delivery outcome				

Mode of delivery (Cesarean VS VBAC)	0.503	1.242 (0.640–2.297)		

Episiotomy	0.278	0.718 (0.387–1.289)		

Perineum/Cervical laceration	0.01	2.128 (1.209–3.808)	0.119	1.715 (0.873–3.411)

Feeding patterns within 48 hours (milk VS breast)	0.169	0.241 (0.013–1.191)		

Feeding amounts within 48 hours (Abnormal VS normal)	0.747	0.814 (0.187–2.500)		

Neonatal weight ≥ 3500	0.086	1.680 (0.916–3.003)		

Admission to neonatal ward after birth	0.324	0.541 (0.127–1.587)		

BMI: body mass index; VAS: visual analog scale; SAS: Self-Rating Anxiety Scale; SSRS: Social Support Rating Scale; MMSE: mini-mental state examination; HGB: hemoglobin; PLT: platelet; ¥: Chinese Yuan. Hosmer and Lemeshow goodness of fit (GOF) test; X-squared (*χ*2) = 8.476, df = 8, *P* value = 0.388. McFadden's pseudo-R squared = 0.206. Cox and Snell pseudo-R squared = 0.157. Nagelkerke pseudo-R squared = 0.279.

**Table 5:** Univariate and multivariate analysis of PPD in TOLAC 48 hours after delivery matched by PSM.

Variable	Univariate (*n* = 214)		Multivariate (*n* = 214)	
Independent	*P* value	OR (95% CI)	*P* value	OR (95% CI)

Epidural analgesia	<0.001	0.257 (0.117–0.528)	0.006	0.327 (0.143–0.708)

Hospital name				
MCH		1 (reference)		
BA	0.18	0.546 (0.210–1.257)		
LG	0.295	0.328 (0.018–1.799)		

General information				
Age (year)	0.782	0.987 (0.896–1.086)		
Gestational age (day)	0.46	1.012 (0.986–1.049)		
BMI (kg/m^2^)	0.684	0.976 (0.864–1.093)		
Maternal education >12 y	0.464	0.771 (0.388–1.571)		
Husband education >12 y	0.104	0.556 (0.276–1.145)		
Housewives	0.699	1.234 (0.385–3.373)		
Family income (¥/mo)^*∗*^				
≤10000		1 (reference)		
10,001–20,000	0.169	0.524 (0.209–1.334)		
>20,000	0.008	0.291 (0.117–0.733)		

History of depression and trauma	0.588	1.953 (0.090–20.855)		

Impact of childbirth on work or re-employment	0.517	1.429 (0.440–3.989)		

Maternity leave time				
No		1 (reference)		
Legal time	0.989	3510585.584 (0.000–NA)		
Full-time	0.988	5397020.941 (0.000–NA)		

Anxiety and depression during pregnancy	0.55	1.228 (0.621–2.398)		

Cigarettes, alcohol, long-term medication	0.337	3.930 (0.153–100.739)		

Whether the husband is satisfied with the baby's sex	0.303	2.975 (0.555–55.127)		

Caring health knowledge during pregnancy				
① Routine obstetric examination	0.24	0.414 (0.098–2.084)		
② Attend maternity classes	0.046	0.483 (0.230–0.968)		
③ Learn about parenting through cell phones or books	0.947	0.972 (0.440–2.324)		

Unplanned pregnancy	0.065	1.891 (0.955–3.724)		

Whether the maternal is the only child in one's family	0.686	1.209 (0.451–2.912)		

Changes in marital relationship during pregnancy	0.337	3.930 (0.153–100.739)		

SAS	<0.001	1.155 (1.082–1.240)	0.003	1.118 (1.041–1.207)

SSRS	<0.001	0.914 (0.868–0.959)	0.01	0.930 (0.879–0.982)

MMSE	0.702	0.943 (0.694–1.274)		
Prepartum Laboratory test				
HGB (g/L: 115–150)	0.77	0.995 (0.963–1.028)		
PLT (125–350)	0.02	0.992 (0.986–0.999)	0.017	0.991 (0.983–0.998)

Initiating lactation period (hours)	0.236	1.019 (0.987–1.050)		

Delivery outcome				

Mode of delivery (Cesarean VS VBAC)	0.337	0.580 (0.164–1.602)		

Episiotomy	0.712	0.878 (0.434–1.730)		

Perineum/Cervical laceration	0.067	1.874 (0.962–3.720)		

Feeding patterns within 48 hours (milk VS breast)	0.988	0.000 (NA–63246344962664952524800406.000)		

Feeding amounts within 48 hours (Abnormal VS normal)	0.696	1.309 (0.281–4.616)		

Neonatal weight ≥ 3500	0.279	1.507 (0.700–3.124)		

Admission to neonatal ward after birth	0.311	0.458 (0.071–1.697)		

PSM: propensity score matching. Data had been matched by using propensity score matching with 1 : 1 nearest neighbor matching. Hosmer and Lemeshow goodness of fit (GOF) test; X-squared (*χ*2) = 3.728, df = 8, *P* value = 0.881. McFadden's pseudo-R squared = 0.192. Cox and Snell pseudo-R squared = 0.177. Nagelkerke pseudo-R squared = 0.278.

**Table 6:** Univariate and multivariate analysis of PPD in TOLAC 42 days after delivery.

Variable	Univariate		Multivariate (*n* = 386)	
Independent	*P* value	OR (95% CI)	*P* value	OR (95% CI)

Epidural analgesia	<0.001	0.210 (0.112–0.380)	<0.001	0.235 (0.113–0.469)

Hospital name				
MCH		1 (reference)		
BA	0.49	0.742 (0.293–1.638)		
LG	0.078	0.337 (0.080–0.970)		

General information				
Age (year)	0.764	1.012 (0.938–1.092)		
Gestational age (day)	0.413	1.012 (0.987–1.045)		
BMI (kg/m^2^)	0.306	0.949 (0.855–1.044)		
Maternal education >12 y	0.714	0.890 (0.484–1.702)		
Husband education >12 y	0.683	0.873 (0.464–1.728)		
Housewives	0.499	1.378 (0.496–3.287)		
Family income (¥/mo)^*∗*^				
≤10,000		1 (reference)		
10,001–20,000	0.45	0.715 (0.307–1.774)		
>20,000	0.26	0.618 (0.275–1.494)		

History of depression and trauma	0.065	6.473 (0.764–54.851)		

Impact of childbirth on work or re-employment	0.309	1.705 (0.547–4.456)		

Maternity leave time				
No		1 (reference)		
Legal time	0.775	0.731 (0.119–14.063)		
Full-time	0.719	1.486 (0.240–28.690)		

Anxiety and depression during pregnancy	0.018	1.979 (1.125–3.487)		

Cigarettes, alcohol, long-term medication	0.35	3.170 (0.146–33.618)		

Whether the husband is satisfied with the baby's sex	0.276	1.963 (0.676–8.341)		

Caring health knowledge during pregnancy				
① Routine obstetric examination	0.881	1.122 (0.303–7.273)		
② Attend maternity classes	0.254	0.713 (0.392–1.264)		
③ Learn about parenting through cell phones or books	0.314	1.581 (0.695–4.270)		

Unplanned pregnancy	0.027	1.891 (1.074–3.329)		

Whether the maternal is the only child in one's family	0.939	0.968 (0.382–2.142)		

Changes in marital relationship during pregnancy	0.038	12.873 (1.214–279.754)		

SAS	0.001	1.079 (1.031–1.130)	0.097	1.051 (0.991–1.114)

SSRS	<0.001	0.922 (0.885–0.961)	0.017	0.945 (0.901–0.990)

MMSE	0.991	0.998 (0.768–1.293)		

Prepartum Laboratory test				
HGB (g/L: 115–150)	0.015	1.027 (1.006–1.051)	0.08	1.027 (0.997–1.058)
PLT (125–350)	0.111	0.996 (0.990–1.001)		

Initiating lactation period (hours)	0.104	1.016 (0.996–1.036)		

Delivery outcome				

Mode of delivery (Cesarean VS VBAC)	0.401	1.325 (0.667–2.503)		

Episiotomy	0.34	0.735 (0.382–1.362)		

Perineum/Cervical laceration	0.015	2.121 (1.166–3.931)	0.076	1.850 (0.942–3.689)

Feeding patterns within 48 hours (milk VS breast)	0.234	1.887 (0.597–5.055)		

Feeding amounts within 48 hours (Abnormal VS normal)	0.474	0.581 (0.091–2.080)		

Neonatal weight ≥ 3500	0.296	1.403 (0.727–2.603)		

Admission to neonatal ward after birth	0.451	0.625 (0.146–1.839)		

Discomfort within 42 days	<0.001	7.692 (4.108–14.466)	<0.001	5.934 (2.823–12.524)

Hosmer and Lemeshow goodness of fit (GOF) test; X-squared (*χ*2) = 10.156, df = 8, *P* value = 0.254. McFadden's pseudo-R squared = 0.230. Cox and Snell pseudo-R squared = 0.162. Nagelkerke pseudo-R squared = 0.302.

**Table 7:** Univariate and multivariate analysis of PPD in TOLAC 42 days after delivery matched by PSM.

Variable	Univariate (*n* = 214)		Multivariate (*n* = 214)	
Independent	*P* value	OR (95% CI)	*P* value	OR (95% CI)
Epidural analgesia	<0.001	0.146 (0.053–0.344)	0.003	0.224 (0.078–0.563)
Hospital name				
MCH		1 (reference)		
BA	0.136	0.465 (0.151–1.179)		
LG	0.391	0.400 (0.021–2.208)		
General information				
Age (year)	0.822	0.988 (0.892–1.095)		
Gestational age (day)	0.582	1.009 (0.983–1.048)		
BMI (kg/m^2^)	0.85	0.988 (0.867–1.115)		
Maternal education >12 y	0.63	0.832 (0.400–1.796)		
Husband education >12 y	0.201	0.611 (0.290–1.329)		
Housewives	0.343	1.688 (0.520–4.712)		
Family income (¥/mo)^*∗*^				
≤10000		1 (reference)		
10,001–20,000	0.305	0.593 (0.221–1.664)		
>20,000	0.082	0.419 (0.159–1.156)		
History of depression and trauma	0.473	2.431 (0.111–26.034)		
Impact of childbirth on work or re-employment	0.565	1.411 (0.382–4.228)		
Maternity leave time				
No		1 (reference)		
Legal time	0.527	0.474 (0.057–9.860)		
Full-time	0.929	0.900 (0.108–18.805)		
Anxiety and depression during pregnancy	0.053	2.027 (0.993–4.185)		
Cigarettes, alcohol, long-term medication	0.266	4.889 (0.190–125.562)		
Whether the husband is satisfied with the baby's sex	0.412	2.386 (0.443–44.292)		
Caring health knowledge during pregnancy				
① Routine obstetric examination	0.717	1.482 (0.253–28.152)		
② Attend maternity classes	0.487	0.773 (0.366–1.584)		
③ Learn about parenting through cell phones or books	0.116	2.409 (0.891–8.431)		
Unplanned pregnancy	0.085	1.885 (0.910–3.879)		
Whether the maternal is the only child in one's family	0.994	0.996 (0.317–2.623)		
Changes in marital relationship during pregnancy	0.266	4.889 (0.190–125.562)		
SAS	<0.001	1.145 (1.069–1.233)	0.03	1.090 (1.010–1.182)
SSRS	0.007	0.933 (0.886–0.980)	0.132	0.957 (0.904–1.014)
MMSE	0.729	0.944 (0.677–1.309)		
Prepartum Laboratory test				
HGB (g/L: 115–150)	0.377	1.015 (0.982–1.049)		
PLT (125–350)	0.706	0.999 (0.992–1.005)		
Initiating lactation period (hours)	0.279	1.016 (0.985–1.046)		
Delivery outcome				
Mode of delivery (Cesarean VS VBAC)	0.653	0.773 (0.216–2.166)		
Episiotomy	0.531	0.790 (0.368–1.631)		
Perineum/Cervical laceration	0.091	1.862 (0.912–3.889)		
Feeding patterns within 48 hours (milk VS breast)	0.195	2.263 (0.586–7.403)		
Feeding amounts within 48 hours (Abnormal VS normal)	0.953	0.954 (0.143–3.824)		
Neonatal weight ≥ 3500	0.563	1.269 (0.545–2.775)		
Admission to neonatal ward after birth	0.199	0.261 (0.014–1.338)		
Discomfort within 42 days	<0.001	7.171 (3.162–16.468)	0.002	4.348 (1.749–10.861)

PSM: propensity score matching. Data had been matched by using propensity score matching with 1 : 1 nearest neighbor matching. Hosmer and Lemeshow goodness of fit (GOF) test; X-squared (*χ*2) = 10.081, df = 7, *P* value = 0.188. McFadden's pseudo-R squared = 0.229. Cox and Snell pseudo-R squared = 0.190. Nagelkerke pseudo-R squared = 0.316.

## Data Availability

The data used to support the findings of this study are included within the article.
